# 

**Published:** 1887-10

**Authors:** 


CONTENTS

VOL. 34 No. 10.

| | PAGE. |
|---|---|
| Our Prospects and Purposes | 217 |
| Prohibition | 218 |
| Natural Gas | 220 |
| Instinct of Animals | 223 |
| Mesmerism Forty Years Ago | 225 |
| Damp Cellars | 229 |
| Treatment of the Hair | 230 |
| Influence of Drugs, &c | 231 |
| Dress | 232 |
| Literary Thefts | 232 |
| Defective Speech | 233 |
| Flesh Diet for Children | 234 |
| A Curiosum | 235 |
| Over the Daisies | 236 |
| Book Notices | 236 |
| Household | 237 |
| Miscellaneous | 239 |

INDEX TO ADVERTISEMENTS OF HALF A PAGE AND OVER.

| | PAGE. |
|---|---|
| Winchester's Hypophosphate | I |
| Hollow Suppositories | II |
| Celerina | III |
| Lactopeptine | IV |
| Castoria | IV |
| Horsford's Acid Phosphate | V |
| Goodyear Rubber Co. | V |
| Crystalline Phosphate | VI |
| Lactated Food | 3d page cover |
| Crystal Pepsin Tablets—2d Page | " |
| New York College of Magnetics | 4th page Cover |

